# Impacts of Road Deicing Application on Sodium and Chloride Concentrations in Philadelphia Region Drinking Water

**DOI:** 10.1029/2021GH000538

**Published:** 2022-02-01

**Authors:** Yuliza D. Cruz, Marissa L. Rossi, Steven T. Goldsmith

**Affiliations:** ^1^ Department of Geography and the Environment Villanova University Villanova PA USA

**Keywords:** roadway deicing agents, drinking water, sodium ingestion, blood pressure, public health

## Abstract

Historical application of roadway deicing agents (e.g., road salt and brines) has led to an increase in sodium and chloride concentrations in surface water over time. Numerous studies have explored the impacts of road salt on freshwater aquatic organisms such as amphibians and benthic macroinvertebrates; however, the public health risk associated with consuming drinking water with elevated sodium has been largely unexplored in the literature. Yet, sodium ingestion, primarily through diet, has been linked to adverse human health conditions, such as hypertension. This study documents weekly sodium and chloride concentrations in municipal tap water from three municipalities within the Philadelphia metropolitan area during winter 2018–2019 (November through March). A late winter peak in sodium and chloride concentrations was observed for all three municipalities immediately following successive snow events coupled with daily high temperatures above 0°C. Among municipalities, mean and peak sodium and chloride concentrations were associated with relatively higher development in upstream areas. Observed sodium concentrations ranged from 1 to 6.4x the USEPA recommended guideline of 20 mg/L for individuals restricted to a total sodium intake of 500 mg/day. Additionally, the contribution of sodium ingestion from water consumption to the recommended daily sodium intake limits for adults ranged from 3.5% to 18.8% for non‐restricted and 4.2%–33.3% for “low salt” (i.e., <1,500 mg/day) diets, respectively. The study results coupled with a records review for 40 U.S. municipalities in snow affected regions indicate the need for real‐time communication between water utilities and the general public regarding sodium exposure risk during winter months.

## Key Points

- Peak tap water sodium and chloride concentrations in winter months coincided with a late season melting event
- Average and peak tap water sodium and chloride concentrations were associated with relatively higher development in upstream areas
- Contribution of tap water sodium to the recommended daily sodium intake limits ingested by adults on low salt diets ranged from 4.2% to 33.3%

## 1 Introduction

Increasing surface water concentrations of sodium and chloride over time have been documented for regions affected by historical applications of roadway deicing agents, such as road salt and brines (Dailey et al., 2014; Interlandi & Crockett, 2003; Kaushal et al., 2005, 2018; Kelly et al., 2008). In streamwater, annual scale peak sodium and chloride concentrations are typically observed during “first flush” or the early portion of storm events or minor snow melting events when dilution is low (Cooper et al., 2014; Gardner & Royer, 2010; Long et al., 2015). Additionally, legacy impacts of road salt application are manifested in summer months during baseflow conditions when contaminated shallow groundwater enters streams (Kaushal et al., 2005; Kelly et al., 2008). The deleterious impacts of elevated chloride concentrations on freshwater aquatic organisms have been well documented, including decreases in species richness and diversity to both benthic macroinvertebrates (Blasius & Merritt, 2002; Williams et al., 2000) and amphibians (Collins & Russell, 2009; Karraker et al., 2008). Yet, the link between exposure to sodium in drinking water and the risk of hypertension for humans in regions of roadway deicing agent application is seldom explored in the literature. This is in despite dietary sodium intake being recognized as a major risk factor for hypertension in children and adults (He & MacGregor, 2009; Malta et al., 2018).

Given sodium ingestion is largely driven by diet, few studies have evaluated the direct impact of sodium concentrations in drinking water on hypertension. Early studies found mixed results, with several documenting a positive statistical relationship between increased sodium in drinking water ingestion and >1 mmHg rise in systolic and/or diastolic blood pressure (SBP and DBP) for U.S. or European communities with municipal water sources (Hallenbeck et al., 1981; Hofman et al., 1980; Tuthill and Calabrese, 1979, 1981), while others observed no statistical difference (Armstrong et al., 1982; Choi, 1984). Yet, the majority of these studies did not control for confounding factors such as diet (beyond a pre‐test 24‐hr exposure) and/or physical activity of the participants. Furthermore, these studies largely focused on adolescent populations. However, two recent studies in Bangladesh found ingestion of drinking water with sodium concentrations >500 mg/L resulted in an approximate >2 mmHg rise in both SBP and DBP, while controlling for diet and physical activity (Khan et al., 2014; Talukder et al., 2016). Finally, a recent meta‐analysis of applicable studies suggested a positive association between sodium ingestion in drinking water and human blood pressure, more consistently for DBP (Talukder et al., 2017). Though the authors recommended the need for additional studies on the topic, particularly for adults.

Despite these potential adverse health effects, the associated exposure risk from municipal drinking water consumption is largely shielded from the public. For example, sodium is not regulated by the US EPA as a primary or secondary contaminant in drinking water; though the agency recommends concentrations in drinking water not exceed 30–60 mg/L to avoid adverse effects on taste, nor exceed 20 mg/L for individuals restricted to a total sodium intake of 500 mg/day (US EPA, 2003). This lack of regulation prevents many municipal water suppliers from publicly documenting sodium concentrations. When the data are reported, sodium values are typically expressed as either an average or range of values with little to no details on the sample size or date(s) of sample collection. This lack of data transparency prevents consumers from understanding their exposure risk, particularly during winter months when a corresponding spike in sodium concentrations is to be expected.

The U.S. Institute of Medicine of the National Academies have established a tolerable upper intake level (TUL) for dietary sodium intake of 2.3 g/day (Institute of Medicine, 2005), while the U.S. National Institutes of Health (NIH)—National Heart Blood and Lung Institute recommends no more than 1.5 g/day for those on a “low sodium” diet (NIH, 2006). Thus, understanding sodium exposure in drinking water risk is particularly important to subsets of the population with a predisposition to hypertension. For example, approximately 40% of Philadelphia, PA residents (total pop: 1,526,006; U.S. Census Bureau, 2020) identify as Black or African American (non‐Hispanic), a subset of the U.S. population with disproportionate hypertension risk (He et al., 1998; Institute of Medicine, 2005; Weinberger et al., 1982).

Although sodium can be added to drinking water during the water treatment process (e.g., sodium hypochlorite [aka chlorine] for disinfection, sodium hydroxide for pH adjustment, and sodium carbonate for water softening), we hypothesize increased roadway deicing application inputs during winter months will exacerbate post treatment sodium concentrations in surface water sourced systems. Thus, we documented weekly sodium and chloride concentrations in municipal drinking water from three homes within the Philadelphia metropolitan region (1971–2015 average snowfall = 49.022 cm/yr) during winter 2018–2019. Each home is located within a different city (Havertown, Pottstown, and Philadelphia, PA) and is serviced by a unique municipal water supply system. Two of the municipalities (Pottstown and Philadelphia, PA) directly draw upon the Schuylkill River as a sole drinking water source, yet are separated by over 60 river kilometers composed of high density land development; thus, affording the opportunity to examine development thresholds on sodium concentrations within the same municipal water supply source. The third municipality (Havertown, PA) receives its water from a mix of surface and deep groundwater. Finally, we tabulated tap sodium in drinking water reporting data from 40 select cities in regions affected by roadway deicing agents throughout the Northeast and Midwestern U.S.

## 2 Study Area Description

**Figure 1:**
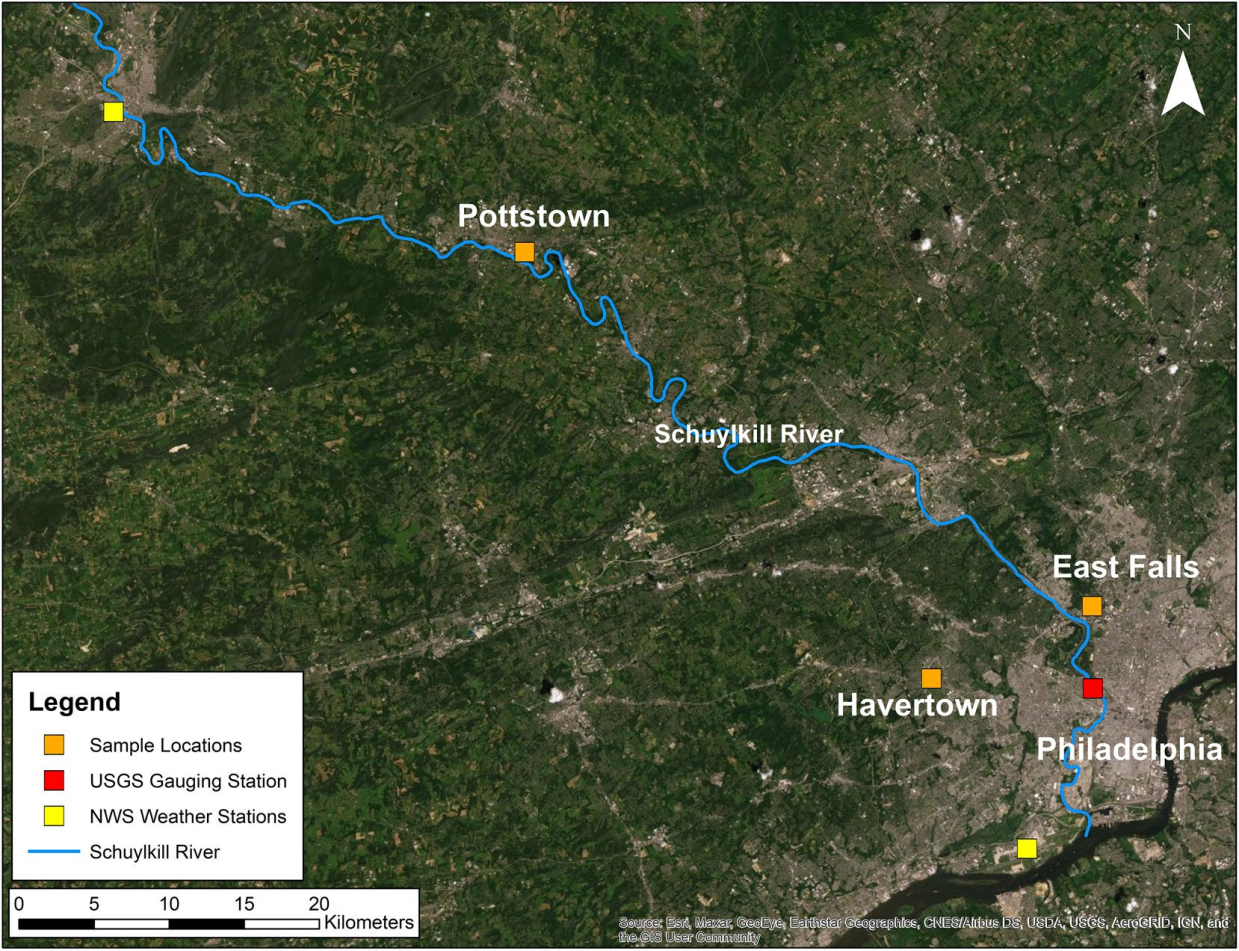
Map of the study area. Municipalities associated with the sample locations are denoted by orange squares. National Oceanic and Atmospheric Administration weather stations are denoted by yellow squares while the United States Geological Survey gauging station is denoted by a red square.

Tap water samples were collected from three single‐family residential homes in southeastern Pennsylvania. Each residence is located within a different municipality (Philadelphia, Pottstown, and Havertown, PA) and serviced by a distinct municipal water supplier (Figure 1). The Philadelphia residence is serviced by the Philadelphia Water Department (PWD). The residence receives water from the Queen Lane reservoir, which draws directly from the Schuylkill River. PWD employs a multi‐step treatment process, which includes the use of sodium hypochlorite for disinfection and both gravity settling and filtration for particulate removal (Philadelphia Water Department [PWD], 2021). PWD maintains publicly available water quality reports (aka Consumer Confidence Reports) documenting annual average and range of sodium concentrations in treated tap water from 2010 to 2020 (PWD, 2011, 2012, 2013, 2014, 2015, 2016, 2017, 2018, 2019, 2020, 2021). Though no data is provided on the number of samples used to obtain these values. Additionally, the PWD does not report chloride data for treated tap water.

The Havertown residence is serviced by Aqua, Inc. The residence is located within the Aqua's Main System (PWSID#: PA1460073), which draws water from the eight surface water sources (Chester, Ridley, Crum, Pickering, Perkiomen, and Neshaminy Creeks, and the Schuylkill River) and a number of groundwater wells (Aqua, 2021). No information regarding the water treatment process or sodium and chloride concentrations in drinking water was found in the 2020 Water Quality Report (Aqua, 2021).

The Pottstown residence is serviced by the Pottstown Borough Authority Water Treatment Plant (PWSID #: 1460037). The Borough Authority draws water directly from the Schuylkill River. No information regarding the water treatment process or sodium and chloride concentrations in drinking water is provided in the 2019 Water Quality Report (Aqua, 2021).

The most recent Water Quality Reports for the Philadelphia and Pottstown municipal systems as well as a Source Water Assessment report for the Havertown system indicate drinking water quality is threatened from roadway deicing agent application (Aqua, 2005; Borough of Pottstown, 2020; PWD, 2021).

## 3 Methods

### 3.1 Water Sample Collection and Analysis

Weekly tap water samples were collected from each of the three residences from 11 November 2018 to 31 March 2019. Samples from the respective residences were usually collected within 1 day of each other. The faucet was run for 30 s prior to collection. Samples were collected in new deionized (DI) water (18 MΩ) washed 500 ml low density polyethylene bottles and subsequently refrigerated. In the laboratory, a 250 ml aliquot of each sample was syringed filtered using 0.63 μm nylon filter and refrigerated until analysis.

Water samples were analyzed for sodium and chloride via ion chromatography (IC) using a Dionex™ DX‐1100 ion chromatograph. While this study focusses on potential human health impacts associated with wintertime variations of sodium in tap water, we also analyzed chloride concentrations to verify road way deicing agents as the causal agent. Precision was determined using five replicate check standards per run with relative standard deviations (RSDs) usually better than ±1% and never greater than ±5% for either. Ion concentrations in DI field and instrument blanks were less than the limit of detection and/or less than 10% of the lowest concentrations in our samples.

### 3.2 Historical Records and Statistical Analysis

Daily and annual temperature and precipitation data for Philadelphia were obtained from the National Oceanic and Atmospheric Administration (NOAA) Philadelphia International Airport site (NOAA Station #: USW00013739; 39° 52ʹ 23.7714″ N, 75° 13ʹ 36.408″ W). Daily temperature data for Reading, Pennsylvania was obtained from the Reading Regional Airport site (NOAA Station #: USW00014712; 40° 22ʹ 24.168″ N, 75° 57ʹ 33.264″ W) while daily precipitation data was sourced from the Reading 2.9 ESE site (NOAA Station #: US1PABR0036; 40° 19ʹ 11.280″ N, 75° 52ʹ 39.720″ W). All present day and historical Water Quality Reports/Consumer Confidence Reports referenced herein were downloaded directly from the municipal agency's website. Finally, daily average discharge and conductivity data for the Schuylkill River at Philadelphia were obtained from the United States Geological Survey (USGS Station# 01474500).

Most statistical calculations, including one‐way analyses of variance (ANOVA), post‐hoc Tukey tests, two‐tailed *t*‐tests were performed using JMP (JMP, 2021). Linear regressions were performed using R software Version 4.0.4 (R Core Team, 2021). Data that did not meet conditions for normality using a Shapiro‐Wilk check test were log transformed prior to analysis.

### 3.3 Sodium in Drinking Water Dosing Equations

For our sodium in drinking water dosing equations, we calculated the percent contribution of sodium in drinking water ingestion to recommended daily sodium intake for adults. Dosing equations (Equations (1), (2), (3), (4)) were established for adults adhering to recommended daily limits as well as those on low salt diets as follows:

(1) (Cmg/L×2.7L/day)/2,300mg/day=%ofdietaryintakeforsodiumindrinkingwateringestion(adultwomen)

(2) (Cmg/L×3.7L/day)/2,300mg/day=%ofdietaryintakeforsodiumindrinkingwateringestion(adultmen)

(3) (Cmg/L×2.7L/day)/1,500mg/day=%ofdietaryintakeforsodiumindrinkingwateringestion–lowsaltdiet(adultwomen)

(4) (Cmg/L×3.7L/day)/1,500mg/day=%ofdietaryintakeforsodiumindrinkingwateringestion–lowsaltdiet(adultmen)

Whereas C refers to the concentration of sodium in a given tap water sample in mg/L. For daily water consumption, we used the Adequate Intake value established by the U.S. Institute of Medicine of the National Academies for an adult male and female of 3.7 and 2.7 L/day, respectively (Institute of Medicine, 2005). For dietary salt intake, we also use the Institute of Medicine's TUL of 2,300 mg/day for the average adult (Institute of Medicine, 2005) and the U.S. NIH‐National Heart Blood and Lung Institute's Dietary Approaches to Stop Hypertension diet recommendation of no more than 1,500 mg/day (aka “low sodium” diet) for populations at risk for hypertension (NIH, 2006). Of note, the American Heart Association (AHA) endorses a maximum threshold of 1,500 mg/day for all healthy adults (AHA, 2021).

## 4 Results

### 4.1 Spatial and Temporal Variations in Concentrations

**Figure 2:**
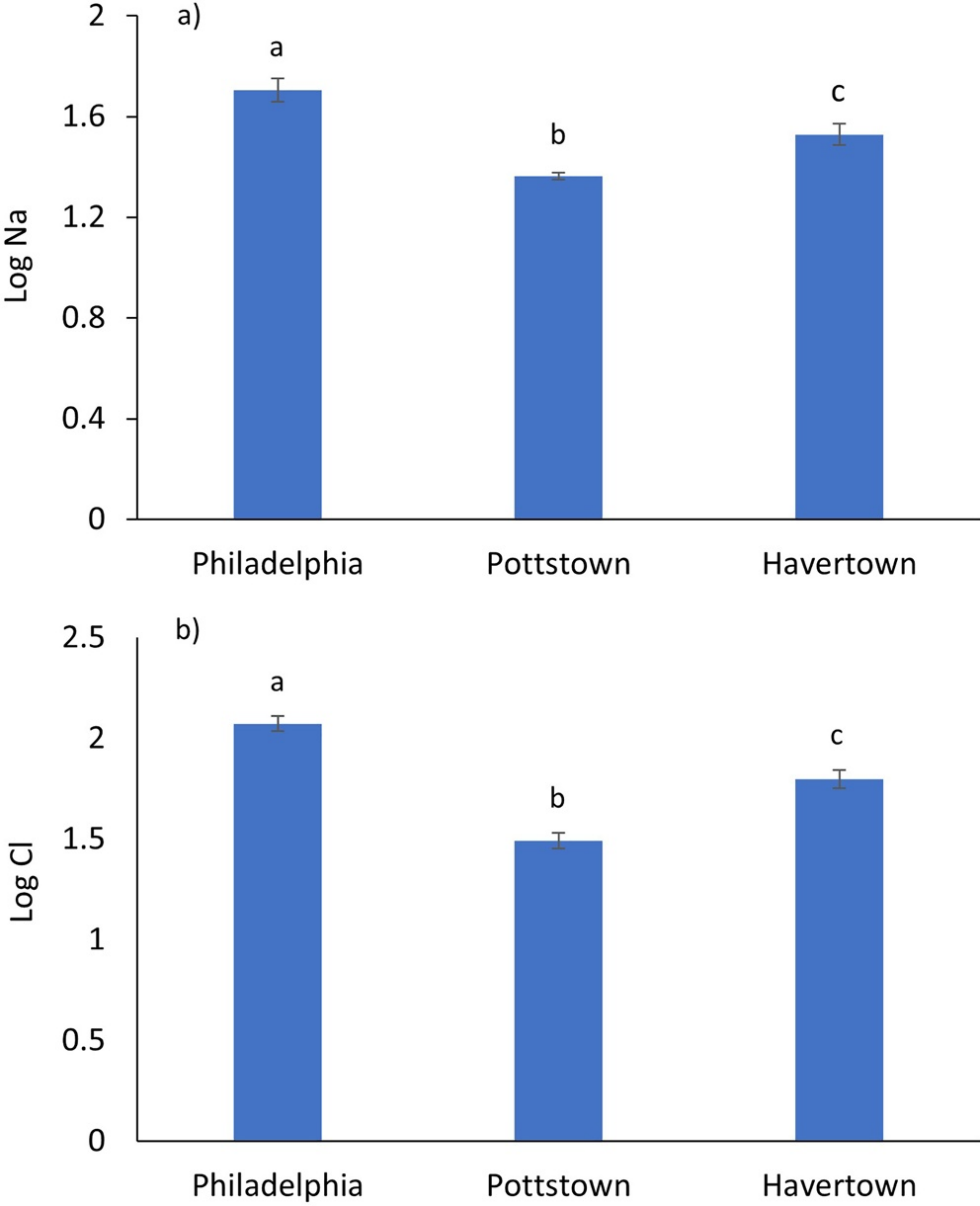
Graphs depicting: (a) mean log Na values of sodium in drinking water for the three study residences, and (b) mean log Cl values of chloride in drinking water for the three study residences. Means that do not share the same letter are statistically significantly different from one another according to an analyses of variance and Tukey HSD post hoc test (*α* = 0.05).

Highest winter mean sodium concentrations were observed for Philadelphia (50.7 mg/L; *n* = 20) followed by Havertown (33.8 mg/L; *n* = 21) and Pottstown (23.1 mg/L; *n* = 19; Table 1). The mean value for Philadelphia was ∼37% greater than the 2019 annual average value (37 mg/L) reported for the Queen Lane water treatment facility by the PWD (PWD, 2020). A series of one‐way ANOVA and Tukey HSD post‐hoc tests (*α* = 0.05) comparing sampling locations revealed statistically significant differences in sodium concentrations for all three municipalities (*p* < 0.05; Figure 2). Peak sodium concentrations for Philadelphia, Pottstown, and Havertown were 127, 32.2, and 76.1 mg/L, respectively. The Philadelphia location also exhibited the largest range (26.5–127 mg/L) and standard deviation (28.7 mg/L).

**Table 1:** Descriptive Statistics for Sodium and Chloride Concentrations (in mg/L) for the Three Residences Examined During This Study During Winter 2018–2019

	Philadelphia (*n* = 20)	Pottstown (*n* = 19)	Havertown (*n* = 21)
*Sodium*			
Mean	50.7	23.1	33.8
Standard deviation	28.7	3.25	18.0
Median	47.4	22.4	29.2
Range (low)	26.5	20.2	19.0
Range (high)	126	32.2	76.1
# of samples in excess of 20 mg/L^a^	20	19	19
*Chloride*			
Mean	118	31.0	62.8
Standard deviation	51.9	13	36.1
Median	113	30.2	53.6
Range (low)	72.7	18	33.7
Range (high)	250	63.8	146
# of samples in excess of 250 mg/L^b^	1	0	0

^a^ US EPA recommendation for sodium in tap water for individuals on severely restricted diets (<500 mg/day).

^b^ US EPA Secondary Drinking Water standard for chloride in tap water to avoid a salty taste (<250 mg/day).

Similar tends were identified for the chloride data. For example, winter mean chloride concentrations were observed in the following order: Philadelphia (118 mg/L; *n* = 20) followed by Havertown (62.8 mg/L; *n* = 21) and Pottstown (31.0 mg/L; *n* = 19; Table 1). A series of one‐way ANOVA and Tukey HSD post‐hoc tests (*α* = 0.05) comparing sampling locations revealed statistically significant differences in chloride concentrations for all three municipalities (*p* < 0.05; Figure 2). The Philadelphia location also exhibited the highest concentration, range (26.5–127 mg/L) and standard deviation (28.7 mg/L).

**Figure 3:**
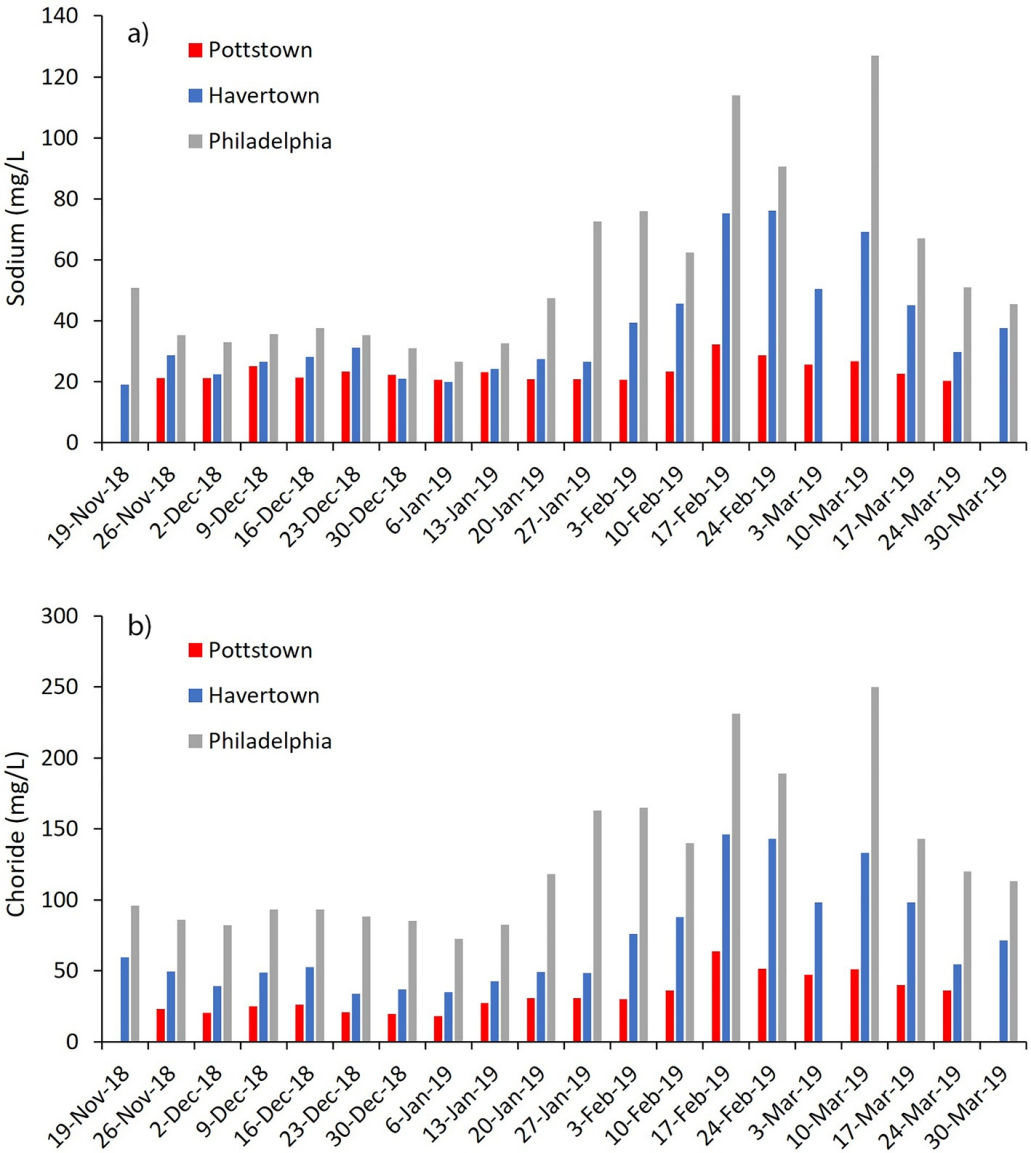
Graphs depicting: (a) weekly sodium concentrations (mg/L) in tap water for each of the three study residences, and (b) weekly chloride concentrations (mg/L) in tap water for each of the three study residences. *Note*. No samples were collected from Pottstown on 19 November 2018 and 30 March 2018, and Philadelphia on 10 March 2019.

**Figure 4:**
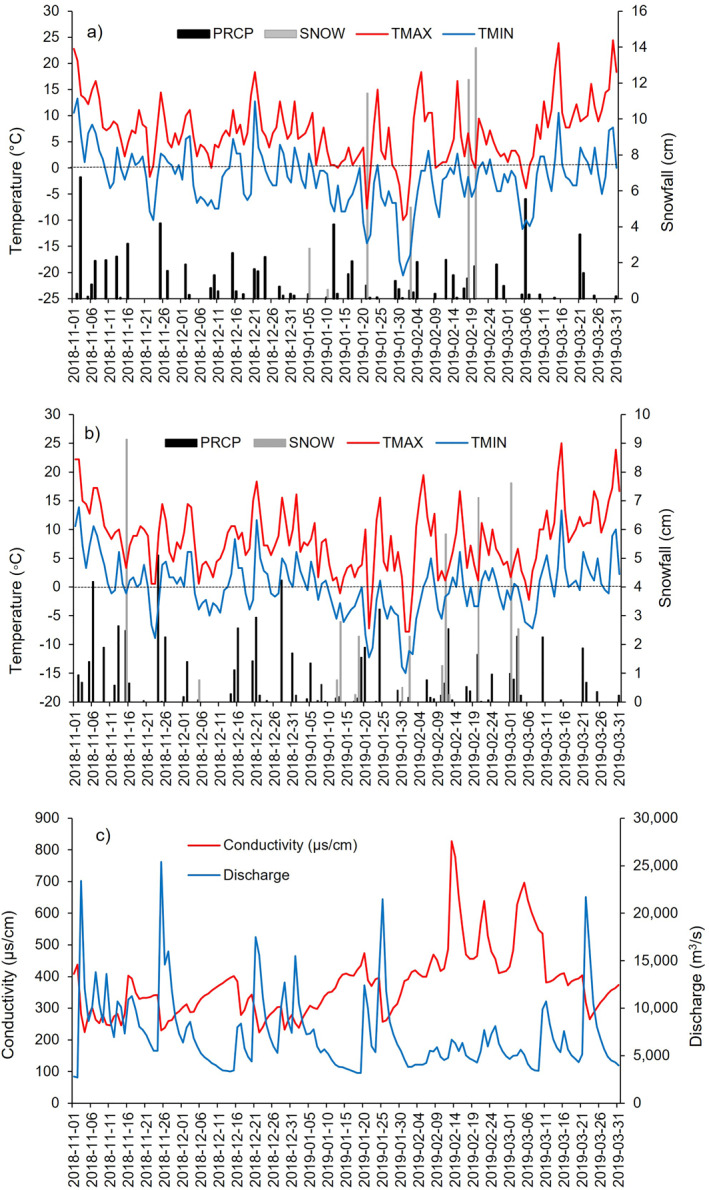
Graphs depicting: (a) daily precipitation (cm), snowfall depth (cm) temperature maximum (°C), temperature minimum (°C) for Reading, PA (NOAA Station #: USW00013739), (b) daily precipitation (cm), snowfall depth (cm) temperature maximum (°C), temperature minimum (cm) for Philadelphia International Airport (NOAA Station #: USW00014712), and (c) average daily discharge data and 5‐day average conductivity values preceding the sampling date for Schuylkill River at Philadelphia (USGS Station #: 01474500).

A relative peak in sodium and chloride concentrations for each sample location was observed from mid‐January through the end of March (Figures 3a and 3b). This temporal peak in concentration coincided with a series of snow events recorded both in the immediate vicinity and upstream of the respective water intakes (Figures 4a and 4b). Additionally, the late season peak concentrations occurred after daily maximum temperatures surpassed 0°C for the remainder of the study period. This temporal trend is confirmed by the positive statistical relationship between the three sites: Philadelphia and Pottstown (Na, *r* ^2^ = 0.46, *p* = 0.003; Cl, *r* ^2^ = 0.82, *p* = < 0.001), Philadelphia and Havertown (Na, *r* ^2^ = 0.78, *p* < 0.001; Cl, *r* ^2^ = 0.80, *p* = < 0.001), and Haverford and Pottstown (Na, *r* ^2^ = 0.72, *p* < 0.001; Cl, *r* ^2^ = 0.90, *p* < 0.001). Interestingly, a strong positive statistical agreement was observed between weekly sodium concentrations for Philadelphia and 5‐day average conductivity values (Figure 4c) preceding the sampling date for the Schuylkill River (USGS Gauging Station #: 01474500; ${r^{2}}_{\text{avg}}$ = 0.71, *p* < 0.001). A similarly strong positive statistical relationship was observed between weekly chloride concentrations and 5‐day average conductivity values for the same location (*r* ^2^ = 0.76, *p* < 0.001).

### 4.2 Long‐Term Data Analysis for Philadelphia

**Figure 5:**
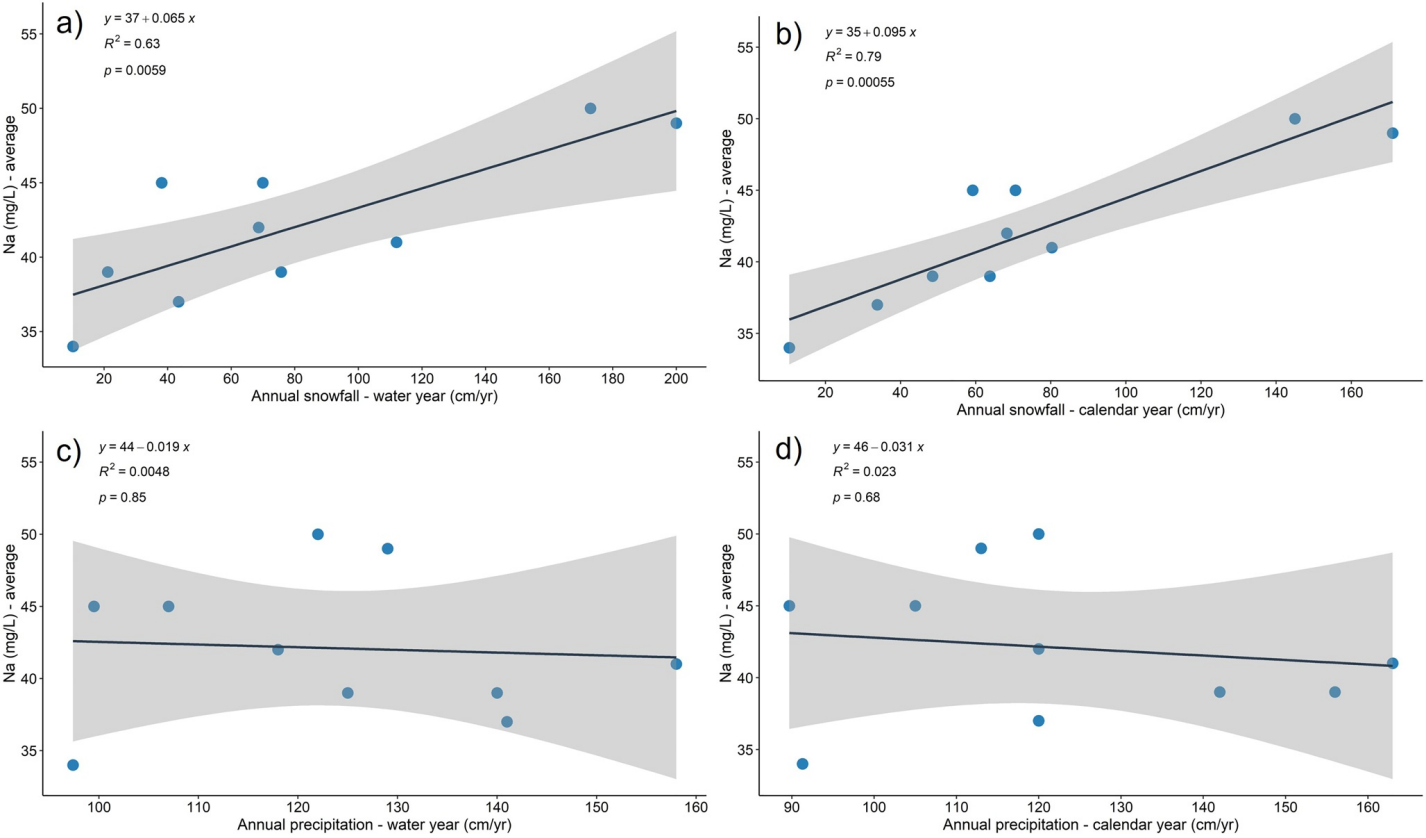
Comparison of National Oceanic and Atmospheric Administration (NOAA) annual precipitation/snowfall data for Philadelphia International Airport (NOAA Station #: USW00014712) and Philadelphia Water Department annual average/high range sodium concentrations (mg/L) in tap water (Queens Lane Reservoir) for the period 2010–2019: (a) annual average sodium concentrations (mg/L) versus water year snowfall totals (cm), (b) annual average range sodium concentrations (mg/L) versus calendar year snowfall totals (cm), (c) annual average sodium concentrations (mg/L) versus water year precipitation totals (cm), and (d) annual average range sodium concentrations (mg/L) versus calendar year precipitation totals (cm).

Positive statistical relationships were observed between annual average in drinking water concentrations in Philadelphia tap water (Queens Lane reservoir) and water year (*r* ^2^ = 0.63, *p* = 0.006) and calendar year snowfall (*r* ^2^ = 0.79, *p* = 0.0006) totals recorded at the Philadelphia Airport (Table 2, Figure 5). Equally strong correlations were observed between annual high range sodium concentrations and water year (*r* ^2^ = 0.65, *p* = 0.005) and calendar year snowfall (*r* ^2^ = 0.63, *p* = 0.006). Conversely, no correlations were observed between average sodium in drinking water concentrations and water year total precipitation (*r* ^2^ = 0.005, *p* = 0.85) or calendar year total precipitation (*r* ^2^ = 0.02, *p* = 0.68).

**Table 2:** Annual Snowfall (cm/yr) and Precipitation (cm/yr) Data, and Annual Average/High Range Sodium Concentrations (mg/L) for Philadelphia, PA From 2010–2019^a^ ^,^ ^b^

					Queen lane		
Year	Annual snowfall—water year (cm/yr)	Annual precipitation—water year (cm/yr)	Annual snowfall—calendar year (cm/yr)	Annual precipitation—calendar year (cm/yr)	Na (mg/L)—average	Na (mg/L)—low	Na (mg/L)—high
2010	200	129	171	113	49	23	115
2011	112	158	80.3	163	41	22	107
2012	10.2	97	10.4	91	34	20	49
2013	21.1	140	48.5	142	39	25	84
2014	173	122	145	120	50	21	111
2015	68.6	118	68.3	120	42	28	65
2016	69.9	99	70.6	90	45	30	60
2017	38.1	107	59.2	105	45	32	72
2018	75.7	125	63.8	156	39	23	63
2019	43.4	141	33.8	120	37	24	80

^a^ Annual snowfall/precipitation data sourced from Philadelphia International Airport (NOAA Station #: USW00013739).

^b^ Annual average and high range sodium data sourced from Philadelphia Water Department, Queens Lane Reservoir (PWD, 2006–2019).

### 4.3 Sodium in Drinking Water Ingestion Risk

The US EPA has a recommended guideline of no more than 20 mg/L of sodium in tap water for individuals on sodium‐restricted diets (<500 mg/day) and an overall recommended threshold of 30–60 mg/L to prevent adverse taste (US EPA, 2003). For the Philadelphia data set (*n* = 20), 100% of samples were above the 20 mg/L threshold (Table 1). In addition, ∼58% (11 out of 19) of samples had concentrations between 30 and 60 mg/L and 37% (7 out of 19) of samples were above 60 mg/L. For Havertown (*n* = 21), ∼90% (19 out of 21) of samples exceeded 20 mg/L, ∼19% (4 out of 21) of samples were between 30 and 60 mg/L, and 14% (3 out of 20) were above 60 mg/L. For Pottstown (*n* = 19), 100% of samples exceeded the 20 mg/L recommendation with only 1 sample falling in the 30–60 mg/L range.

Assuming recommended daily water ingestion criteria are met, the average sodium in drinking water concentration for Philadelphia (56.4 mg/L) would contribute 6.1% toward the recommended TUL (aka daily sodium intake of 2,300 mg) for adult women and 8.3% for adult men (Table 3). Unsurprisingly, these contributions increase for those on sodium restricted diets (e.g., 1,500 mg/day; 10.1% for women and 13.9% for men). However, sodium in drinking water contribution toward the TUL more than doubles on days when there is a spike in concentrations. For instance, substituting the highest sodium in drinking water concentration for Philadelphia almost doubles the relative contribution to the TUL (13.7% for women and 18.8% for men) and for those on low salt diets (22.9% for women and 31.3% for men). For Havertown, average sodium in drinking water concentrations would contribute 4% toward the TUL for adult women and 5.5% for adult men. These percentages substantially increased for peak sodium in drinking water concentrations (8.2% for women and 11.3% for men), particularly with respect to those on low sodium diets (13.7% for women and 18.8% for men). For Pottstown, sodium in drinking water contributions toward the TUL and sodium restricted diets remained relatively low (i.e., <8%).

**Table 3:** Percent Contribution of Water Ingested Sodium Toward Recommended Sodium Ingestion Guidelines

	Tolerable upper limit (TUL)^a^		Low sodium^b^	
	Women (%)	Men (%)	Women (%)	Men (%)
Philadelphia				
Average	6.1	8.3	10.1	13.9
High range	13.7	18.8	22.9	31.3
Havertown				
Average	4.0	5.5	6.7	9.2
High range	8.2	11.3	13.7	18.8
Pottstown				
Average	2.5	3.4	4.2	5.7
High range	3.5	4.8	5.8	7.9

^a^ U.S. Institute of Medicine's tolerable upper limit (TUL) of 2,500 mg day^−1^ (Institute of Medicine, 2005).

^b^ U.S. National Institute of Health‐National Heart blood and Lung Institute's Dietary Approaches to Stop Hypertension diet recommendation of no more than 1.5 g per day for populations at risk of hypertension (NIH, 2006).

## 5 Discussion

### 5.1 Links Between Roadway Deicing Agent Application and Sodium and Chloride in Drinking Water Concentrations in Tap Water

Differences in the magnitude and timing of sodium and chloride peaks between the three residences could be attributed to relative differences in deicing agent application, water sources, and winter storm trajectories. For example, mean sodium and chloride concentrations for Philadelphia were ∼2.2x and 3.8x; greater than those for Pottstown, respectively, despite both municipalities solely drawing water from the Schuylkill River. However, the respective intakes are separated by over 60 river kilometers with a drainage area characterized by high density land development. For example, the majority of Montgomery County, PA, which primarily drains into the Schuylkill River watershed, is located between the two intakes. Montgomery County is the third most populous county in the state (pop: 830, 915; 2019 estimate) and has the third highest population density (1,261.32 people per km^2^; U.S. Census Bureau, 2020). Conversely, the water distribution system for Havertown draws upon water from municipal groundwater wells in addition to river sources. A previous analysis of sodium concentrations in groundwater wells for the region found sodium concentrations were predominantly less than 30 mg/L with the majority falling within in the 5–10 mg/L range (Senior et al., 1997). Therefore, any mixing of municipal water with a groundwater source is likely to lead to a dilution effect.

A late season peak in sodium and chloride concentrations for all three sample locations was immediately preceded by snow fall events and/or increasing daily maximum temperatures (Figures 2 and 4). The causation of roadway deicing agent application as the source of this trend is supported by a two‐fold increase in streamwater conductivity for the Schuylkill River over this same period, as well as the strong link between chloride and conductivity in similarly affected regions (Haq et al., 2018). Similarly, Leslie and Lyons (2018) attributed a winter‐time spike of chloride concentrations in Columbus, Ohio tap water to the application of roadway deicing agents. Furthermore, winter‐time seasonal peaks in sodium and/or chloride concentrations have also been observed in surface water bodies used for drinking water purposes throughout the northeastern and midwestern United States (Dailey et al., 2014; Kaushal et al., 2018, 2021; Kelly et al., 2008).

Peak sodium concentrations for the Philadelphia sampling location were amongst the highest documented for Philadelphia tap water (Table 2; PWD, 2011, 2012, 2013, 2014, 2015, 2016, 2017, 2018, 2019, 2020) as well ∼40 municipalities in the northeast and north‐central United States (Table 4). Though a lack of information regarding the timing and frequency of sample collection from the associated municipal reports suggests absolute sodium concentrations could be much higher than what has been reported. Interestingly, the 2018–2019 winter snowfall total recorded at the Philadelphia Airport (17.1 cm) was well below the 1971–2015 long‐term average (49.02 cm/yr). This relative lack of snowfall during the study period suggests peak tap water sodium concentrations for all three sampling locations could also be much higher in years with elevated snowfall. Additionally, our weekly sampling protocol may have also missed peak sodium concentration during the late season meltwater event.

**Table 4:** Tap Water Sodium Data (in mg/L) From Select Cities Throughout the Northeast and Midwestern U.S.^a^

City, state	Average sodium concentration (mg/L)	Range (mg/L)	Drinking water source	Year of record	# Of samples collected provided (Y/N)^b^	Language on risks of sodium ingestion is provided (Y/N)^c^	Recommended value(s) provided for individuals on salt restircitve diets (Y/N)^d^	Acknowledge road salt as potential contaminant (Y/N)^e^		Source
								Indirect	Direct	
*Connecticut*										
Bridgeport, CT	20.3	17.4–37.4	Surface water and groundwater	2020	N	Y	Y	Y	Y	Aquarion (2021a)
Hartford, CT	10.6	7.9–14.4	Surface water	2020	N	Y	Y	N	N	Metropolitan District (2021)
New Haven, CT	20.66	4.7–43.7	Surface water and groundwater	2020	N	Y	Y	Y	Y	Regional Water Authority (2021)
Stamford, CT	37.3	17.4–39.6	Surface water	2020	N	Y	Y	Y	Y	Aquarion (2021b)
*Illinois*										
Chicago, IL	–	8.73–9.55	Surface water	2020	N	Y	N	Y	N	City of Chicago Department of Water Management (2021)
*Indiana*										
Fort Wayne, IN	–	9.6–30	Surface water	2020	N	N	N	Y	N	Fort Wayne City Utilities (2021)
Indianapolis, IN	42	6.8–140	Surface water and groundwater	2020	N	N	N	N	N	Citizens Energy Group (2021)
*Maine*										
Portland, ME	8.7*	–	Surface water	2020	N	N	N	Y	Y	Portland Water District (2021)
Massachusetts										
Boston, MA	–	42.7**	Surface water	2020	N	Y	Y	N	N	Massachusetts Water Resources Authority (2021)
Lowell, MA	33.0*	–	Surface water	2019	N	N	N	Y	Y	Lowell Regional Water Utility (2021)
Springfield, MA	–	13.7**	Surface water	2020	N	N	N	Y	N	Springfield Water and Sewer Commission (2021)
Worchester, MA	–	15**	Surface water	2020	N	Y	Y	Y	Y	City of Worcester Water Operations (2021)
*Michigan*										
Detroit, MI	5.14	4.43–7.78	Surface water	2020	N	N	N	Y	N	City of Detroit Water and Sewerage Department (2021)
Grand Rapids, MI	11*	–	Surface water	2020	N	N	N	Y	N	City of Grand Rapids (2021)
*New Hampshire*										
Portsmouth, NH	74	22–180	Surface water	2020	N	N	N	Y	N	City of Portsmouth Department of Public Works (2021)
Nashua, NH	47.2*	–	Surface water	2020	N	N	N	Y	Y	Pennichuck Corporation (2021)
*New Jersey*										
Elizabeth, NJ	–	18–101	Surface water and groundwater	2020	N	Y	N	N	N	New Jersey American Water (2021)
Newark, NJ	23.2*	–	Surface water	2018	N	N	N	Y	Y	Newark Water and Sewer (2019)
Jersey City, NJ	–	36–60	Surface water	2020	N	Y	Y	Y	N	Suez (2021)
Paterson, NJ	–	46.1–94.8	Surface water	2020	N	Y	Y	Y	Y	Passaic Valley Water Commission (2021)
*New York*										
Albany, NY	20.3	20.0–270	Surface water	2020	*N*	Y	Y	Y	Y	City of Albany Department of Water and Water Supply (2021)
Buffalo, NY	11	–	Surface water	2020–2021	N	Y	Y	Y	Y	Buffalo Water (2021)
New York City, NY	12	9–44	Surface water	2020	Y (*n* = 300)	Y	Y	Y	Y	New York City Department of Environmental Protection (2021)
Rochester, NY^f^	15	14–16	Surface water	2020	N	N	N	Y	Y	City of Rochester Department of Environmental Services (2021)
Syracuse, NY^f^		17.8–18.9	Surface water	2019	N	Y	Y	Y	Y	City of Syracuse Department of Water (2021)
Yonkers, NY	–	10.5–12.2	Surface water	2020	N	Y	Y	Y	Y	City of Yonkers Bureau of Water (2021)
*Ohio*										
Akron, OH	201*	–	Surface water	2020	N	N	N	Y	N	Akron Water Supply Bureau (2021)
Cleveland, OH	10.4	–	Surface water	2020	N	N	N	Y	N	Cleveland Water (2021)
Cincinnati, OH	–	25–32	Surface water	2020	Y	N	N	Y	N	Greater Cincinnati Water Works (2021)
Columbus, OH^g^	48.6	21.0–97.6	Surface water	2020	N	N	N	Y	N	City of Columbus Department of Public Utilities (2021)
Toledo, OH	–	9.1–22.8	Surface water	2020	N	Y	Y	Y	N	City of Toledo (2021)
*Pennsylvania*										
Philadelphia, PA^h^	37	26–44	Surface water	2020	N	N	N	N	N	PWD (2021)
Pittsburgh, PA	–	–	Surface water	2020	N/A	N	N	Y	N	Pittsburgh Water and Sewer Authority (2021)
Harrisburg, PA	–	–	Surface water	2020	N/A	N	N	Y	N	Capitol Region Water (2021)
Reading, PA	–	–	Surface water	2020	N/A	N	N	Y	N	Reading Area Water Authority (2021)
Scranton, PA	–	16–201	Surface water and groundwater	2020	N	Y	Y	Y	Y	Pennsylvania American Water (2021)
*Rhode Island*										
Providence, RI	15*	–	Surface water	2020	N	N	N	Y	Y	Providence Water (2021)
*Vermont*										
Burlington, VT	20*	–	Surface water	2020	N	N	N	Y	Y	Burlington Water Resources (2021)
*Wisconsin*										
Green Bay, WI	8.3*	–	Surface water	2020	N	N	N	Y	N	Green Bay Water Utility (2021)
Milwaukee, WI	–	9.6–9.7	Surface water	2020	N	N	N	Y	Y	Milwaukee Water Works (2021)

*Note*. –, No data provided; *, Data presented as detected value rather than average value; **, Data presented as highest recorded value.

^a^ The 40 selected cities maintain municipal water systems which serve populations greater than 40,000 individuals and utilize surface water as a predominant source. This table does not include all cities which meet these criteria in the aforementioned states.

^b^ The number of samples used to determine average water sodium concentrations or range of values is specifically stated in the Water Quality or Consumer Confidence Report.

^c^ Language on risks of sodium ingestion is provided, particularly with respect to those on sodium restricted diets.

^d^ Specific USEPA recommended water sodium concentrations are provided for those on moderately and severely restricted sodium diets.

^e^ Either USEPA recommended langugage acknowledging "salts" as one of many contaminants which can enter stormwater runoff (indirect) or a specific acknowledgment of road salt as a contaminant (direct) is provided.

^f^ Data for lake Ontario, only.

^g^ Data from Dublin Road Water Treatment Plant, only.

^h^ Data taken from East Falls Reservoir, only.

The positive statistical relationship between annual average tap water sodium concentrations with water year snowfall depth for Philadelphia, suggests roadway deicing agents are responsible for variations on annual time scales. This mode of causation was further supported by the lack of statistical relationship between average annual tap water sodium concentrations with either water year total precipitation or calendar year total precipitation. Increased sodium hypochlorite additions that may occur in response to increased surface water turbidity or combined sewage overflow at the water treatment plant prior to distribution are an unlikely driver of the sodium concentrations we report.

To confirm this relationship between annual snowfall and sodium concentrations in tap water, we repeated this analysis for the City of Columbus, OH, another snow affected region with a 10+ year availability of CCRs that document average annual sodium levels (Figure S1 and Table S2 in Supporting Information S1). For this locale, we used data for the Dublin Road Water Plant; the only city drinking water plant solely sourced by surface water. We observed a positive, near statistically significant relationship between average annual tap water sodium concentrations with water year annual snowfall data recorded at the John Glenn International Airport (NOAA Station#: US1OHFR0052; *r* ^2^ = 0.28, *p* = 0.052) and a near statistically significant relationship with calendar year snowfall (*r* ^2^ = 0.32, *p* = 0.035). Again, these relationships reduced in strength when annual average sodium concentrations were compared with either water year total precipitation (*r* ^2^ = 0.17, *p* = 0.14) or calendar year total precipitation (*r* ^2^ = 0.18, *p* = 0.13). This analysis further suggests that snowfall totals could be used as a predictor of tap water sodium risk.

Annual roadway deicing agent application may also provide chronic stress to tap water sodium concentrations. For example, an increase in sodium and chloride concentrations over time has been observed in surface water bodies utilized for drinking water throughout snow affected regions in the northeastern and north‐central United States (Dailey et al., 2014; Kaushal et al., 2005, 2018). Similarly, a long‐term (1973–1999) analysis of USGS and PWD water samples drawn from the Schuylkill River found a significant increase in annual sodium concentrations (0.321 mgl/yr, *r* ^2^ = 0.3252, and *p* = 0.0024) with annual average values ranging from ∼12 to 30 mg/L (Interlandi & Crockett, 2003). Interestingly, average tap water sodium concentration reported for the Queens Lane water treatment plant appear to have stabilized from 2010 to 2019 (−0.35 mgl/yr, *r* ^2^ = 0.04, and *p* = 0.58), which corresponds with a non‐statistically significant decrease in recorded snowfall over the same period (−6.76 cm/yr, *r* ^2^ = 0.18, and *p* = 0.22) recorded at the Philadelphia International Airport (Table 2). A climate model predicting a decrease in future snowfall for southeast Pennsylvania of 50%–60% from current values may alleviate this chronic stress in future decades (Kapnick & Delworth, 2013a, 2013b).

### 5.2 Assessing Sodium Exposure Risk

Tap water sodium concentrations from all three homes were of immediate risk to those on severely restricted diets (<500 mg/day). Furthermore, the relative risk to those with non‐restrictive (2,300 mg/day) and low‐salt diets (1,500 mg/day) dramatically increased during the meltwater event that occurred toward the end of the study period. For example, high‐range sodium concentrations in drinking water for Philadelphia substantially contributed toward total sodium intake for normal and low salt diets (∼14% to 18% and ∼23% to 31%, respectively. Yet, the 2018–2019 winter for the region was characterized by well below average snowfall. Furthermore, our weekly sampling regimen likely missed true peak sodium concentrations and the extent to which sodium values remained highly elevated. Thus, observed values are likely an underestimate of true wintertime sodium in drinking water exposure risk for the region.

Several studies have documented the positive relationship between dietary sodium ingestion (estimated via urinary sodium excretion) and blood pressure with increasing effects at higher intake levels (He & MacGregor, 2009; Lamelas et al., 2016). From a sodium in drinking water ingestion perspective, early studies documented blood pressure differences in communities with concentrations as low as 107 mg/L (Tuthill & Calabrese, 1979), a value in line with the highest individual values from the Philadelphia residence in this study. More recently, a study evaluating the link between sodium ingestion in drinking water and blood pressure in regions impacted by saltwater intrusion in coastal Bangladesh, found a 0.1 g increase in water salinity concentration contributes to a 0.22 mmHg increase in SBP (Talukder et al., 2016). These findings suggest temporary spikes in water concentrations are of particular concern to vulnerable subsets of the population with a predisposition to hypertension. In the case of Philadelphia, ∼40% of Philadelphia, PA residents identify as Black or African American (non‐Hispanic), a subset of the U.S. population with disproportionate hypertension risk (He et al., 1998; Institute of Medicine, 2005; Weinberger et al., 1982).

Finally, many studies have documented a seasonal increase in the presentation of hypertension and cardiovascular diseases (i.e., heart attack, stroke, etc.) during winter months (Fares, 2013; Hong et al., 2003; Takenaka et al., 2010; Thomas et al., 2008). For hypertension, seasonal peaks have been attributed to a variety of factors, including but not limited to outdoor temperature, physical activity, seasonality of vitamin D, seasonal variation in serum cholesterol and other factors (Fares, 2013). It follows that a greater propensity for hypertension increases the manifestation of both renal and heart complications (Johnson et al., 2005; Petrie et al., 2018). Our results suggest elevated sodium concentrations in drinking water during this same seasonal time frame could be a confounding factor for this seasonal presentation of hypertension and related adverse health conditions. Future studies evaluating the timing of peak tap water sodium concentrations and the manifestation of cardiovascular events in hospital emergency rooms may elucidate this potential relationship. Health care professionals responsible for patients with hypertension, renal impairments and cardiovascular disease could also promote the use of bottled water in their patients during peak periods.

### 5.3 The Need for Increased Transparency From Municipal Water Utilities

The study results point to the need for increased transparency between water utilities and the general public in regions where roadway deicing agents are applied; both with respect to sampling frequency and temporal spikes in sodium concentrations during winter months. For example, our review of City of Philadelphia Water Quality Reports for calendar years 2010–2020 yielded no information regarding the timing of samples collected for the determination of average and range of sodium concentrations. Additionally, no information was provided regarding exceedances of the US EPA threshold of 20 mg/L for those on severely restricted sodium diets. While water utility public outreach efforts have traditionally focused on water quality issues, such as taste, color, and concentration of lead and inorganic contaminants (Heath, 2018; Sweeney, 2020), real‐time knowledge regarding sodium risk would allow consumers to take preventive measures.

To illustrate this need for increased transparency from water utilities, we tabulated sodium reporting data from publicly available Water Quality and/or Consumer Confidence Reports for 40 cities in snow affected regions throughout the northeastern and midwestern United States (Table 4). In general, the selected cities serve populations greater than 40,000 individuals and utilize surface water as a predominant drinking water source; though not all cities that meet these criteria in the aforementioned states are summarized herein. Data were compiled for the 2020 calendar year with the exception of two cities.

Average treated tap water sodium concentrations are reported for ∼33% (13 out of 40) of the cities. Additionally, 25% (10 out of 40) of the cities provide a “detected value” while three others provide the highest recorded value. A total of 55% (22 out of 40) of the cities provide a range of values in treated water. Interestingly, only one municipality clearly indicated the number of water samples used to determine these aforementioned values (e.g., New York City, *n* = 300; New York City Department of Environmental Protection, 2021).

Information regarding potential impacts to human health was equally limited. For example, only 43% (17 of 40) of the reports indicated some relationship between tap water sodium ingestion and human health. An equally limited number of reports ∼38% (15 out of 40) provided guidance values for sodium that could impact human health. For example, a 2020 Water Quality Report for Albany, New York stated “Water containing more than 20 ppm of sodium should not be used for drinking by people on diets that severely restrict sodium. Water containing more than 70 ppm of sodium should not be used by people on diets that moderately restrict sodium.” (City of Albany Department of Water & Water Supply, 2021). Yet, none of these reports indicated the extent to which these human health thresholds were surpassed during the calendar year, thus providing little benefit to susceptible populations.

Finally, we observed substantial differences in language regarding the causation of relative water concentrations. For example, only 48% (19 out of 40) of the municipalities list either “road salts” or “roadway deicing agents” as a direct source of observed sodium concentrations. Conversely, 88% (35 out of 40) of the reports included the following US EPA recommended language, which indirectly attributes urban stormwater runoff for concentrations of salts in drinking water: “Inorganic contaminants, such as salts and metals, which can be naturally occurring or result from urban storm water runoff, industrial or domestic wastewater discharge, oil and gas production, mining or farming” (US EPA, 2021). Regardless, these statements provide additional affirmation regarding the link between roadway deicing agent application and sodium concentrations in drinking water.

## 6 Conclusions

This study is one of the first to document a relationship between snowfall and sodium and chloride concentrations in drinking water for regions that rely on surface water as a primary drinking water source. Peak tap water sodium and chloride concentrations in all three sample locations corresponded with a late season snowfall/snowmelt period. Statistically distinct sodium and chloride concentrations were identified for each of the three sampling locations, with Philadelphia exhibiting the highest overall values. Contribution of tap water sodium ingestion to recommended daily sodium intake limits for adults ranged from 3.5% to 18.8% for non‐restricted and 4.2%–33.3% for low salt diets, respectively. Additionally, almost all of the samples for the three municipalities exceeded the 20 mg/L threshold for those on severely restricted sodium diets. Finally, a records review of water quality/consumer confidence reports for 40 municipalities confirms the need for both increased wintertime sampling for sodium as well as greater transparency regarding the timing of spikes in tap water sodium concentrations in regions where roadway deicing agents are applied.

## Conflict of Interest

The authors declare no conflicts of interest relevant to this study.

## Supporting information

Supporting Information S1

## Data Availability

All tap water data used in this study has been uploaded to the Harvard Dataverse (Goldsmith et al., 2022): https://doi.org/10.7910/DVN/RX6515.
