# Supplementary material for: Impacts of Road Deicing Application on Sodium and Chloride Concentrations in Philadelphia Region Drinking Water
Source: Geohealth. 2022 Feb 1;6(2):e2021GH000538. doi: 10.1029/2021GH000538 (PMC8859511; doi:10.1029/2021GH000538)
Supplement: Supplementary file 1 — Supporting Information S1 [file GH2-6-e2021GH000538-s001.docx]

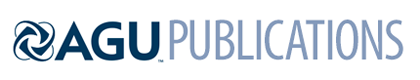


*GeoHealth*

Supporting Information for

**Impacts of road deicing application on sodium and chloride concentrations in Philadelphia region drinking water**

Yuliza D. Cruz^1^, Marissa L. Rossi^1^, and Steven T. Goldsmith^1^

Department of Geography and the Environment, Villanova University, Villanova, PA 19085, USA

**Contents of this file**

Figure S1

Tables S1 to S2

Data Set S1


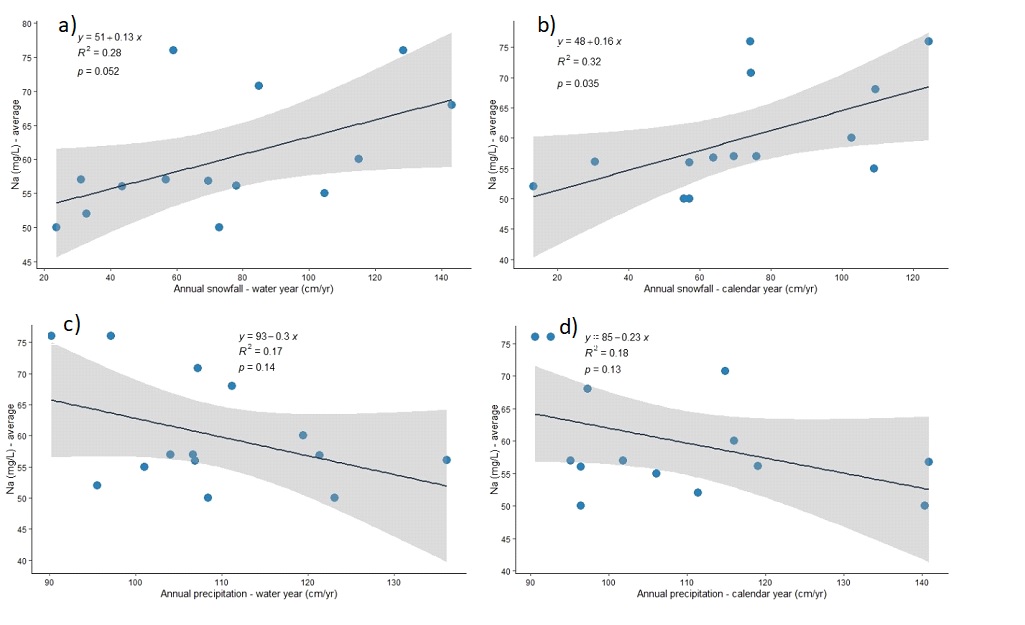


Figure S1. Comparison of NOAA annual precipitation/snowfall data for John Glenn International Airport (NOAA Station #: US1OHFR0052) and City of Columbus Water Department annual average/high range sodium concentrations (mg/L) in tap water (Dublin Road Water Plant) for 2006-2019: a) Annual average sodium concentrations (mg/L) vs. water year snowfall totals (cm), b) Annual average sodium concentrations (mg/L) vs. calendar year snowfall totals (cm), c) Annual high range sodium concentrations (mg/L) vs. water year total precipitation (cm), d) Annual high range sodium concentrations (mg/L) vs. calendar year total precipitation (cm).

| Philadelphia | |  | Pottstown | |  | Havertown | |  |
| --- | --- | --- | --- | --- | --- | --- | --- | --- |
| Sample Date | Na (mg/L) | Cl (mg/L) | Sample Date | Na (mg/L) | Cl (mg/L) | Sample Date | Na (mg/L) | Cl (mg/L) |
| 19-Nov-18 | 50.7 | 95.9 | 18-Nov-18 | NS | NS | 18-Nov-18 | 19.0 | 59.3 |
| 26-Nov-18 | 35.3 | 86.1 | 25-Nov-18 | 21.2 | 23.0 | 25-Nov-18 | 28.7 | 49.7 |
| 2-Dec-18 | 32.9 | 82.2 | 2-Dec-18 | 21.2 | 20.3 | 2-Dec-18 | 22.4 | 39.1 |
| 9-Dec-18 | 35.6 | 93.2 | 9-Dec-18 | 25.1 | 25.2 | 9-Dec-18 | 26.4 | 48.7 |
| 16-Dec-18 | 37.6 | 93.1 | 16-Dec-18 | 21.4 | 26.1 | 16-Dec-18 | 28.1 | 52.6 |
| 23-Dec-18 | 35.3 | 88.2 | 23-Dec-18 | 23.2 | 20.7 | 23-Dec-18 | 31.1 | 33.7 |
| 30-Dec-18 | 31.0 | 85.2 | 30-Dec-18 | 22.3 | 19.6 | 30-Dec-18 | 21.2 | 36.7 |
| 6-Jan-19 | 26.5 | 72.7 | 6-Jan-19 | 20.5 | 18.0 | 6-Jan-19 | 19.9 | 35.0 |
| 13-Jan-19 | 32.5 | 82.4 | 13-Jan-19 | 23.1 | 27.4 | 13-Jan-19 | 24.1 | 42.5 |
| 20-Jan-19 | 47.4 | 118 | 20-Jan-19 | 20.8 | 30.7 | 20-Jan-19 | 27.4 | 49.1 |
| 27-Jan-19 | 72.6 | 163 | 27-Jan-19 | 20.8 | 30.6 | 27-Jan-19 | 26.5 | 48.5 |
| 3-Feb-19 | 75.9 | 165 | 3-Feb-19 | 20.6 | 29.8 | 3-Feb-19 | 39.3 | 76.0 |
| 10-Feb-19 | 62.4 | 140 | 10-Feb-19 | 23.4 | 36.2 | 10-Feb-19 | 45.6 | 87.9 |
| 17-Feb-19 | 114 | 231 | 17-Feb-19 | 32.2 | 63.8 | 17-Feb-19 | 75.1 | 146 |
| 24-Feb-19 | 90.5 | 189 | 24-Feb-19 | 28.6 | 51.4 | 24-Feb-19 | 76.1 | 143 |
| 3-Mar-19 | NS^1^ | NS | 3-Mar-19 | 25.6 | 47.1 | 3-Mar-19 | 50.4 | 98.0 |
| 10-Mar-19 | 127 | 250 | 10-Mar-19 | 26.7 | 51.2 | 10-Mar-19 | 69.2 | 133 |
| 17-Mar-19 | 67.0 | 143 | 17-Mar-19 | 22.5 | 40.1 | 17-Mar-19 | 45.1 | 98.0 |
| 24-Mar-19 | 51.0 | 120 | 24-Mar-19 | 20.2 | 36.0 | 24-Mar-19 | 29.7 | 54.6 |
| 30-Mar-19 | 45.3 | 113 | 31-Mar-19 | NS | NS | 31-Mar-19 | 37.5 | 71.5 |
| ^1^NS = No sample collected on given date | | |  |  |  |  |  |  |

Table S1. Sodium and chloride concentrations (mg/L) in tap water for three residences sampled weekly during winter 2018-2019

|  |  |  |  |  | Dublin Road Water Treatment Plant | | |
| --- | --- | --- | --- | --- | --- | --- | --- |
|  | Annual snowfall- water year (cm/yr) | Annual precipitation - water year (cm/yr) | Annual snowfall- calendar year (cm/yr) | Annual precipitation- calendar year (cm/yr) | Na (mg/L) - Average | Na (mg/L) - Low | Na (mg/L) - High |
| 2006 | 32.8 | 95.6 | 13.2 | 111 | 52 | 38 | 75 |
| 2007 | 56.6 | 107 | 76 | 102 | 57 | 30 | 94 |
| 2008 | 115 | 119 | 103 | 116 | 60 | 38 | 111 |
| 2009 | 58.9 | 90.3 | 74.2 | 90.6 | 76 | 54 | 98 |
| 2010 | 129 | 97.1 | 124 | 92.6 | 76 | 55 | 120 |
| 2011 | 72.9 | 123 | 55.6 | 140 | 50 | 20 | 161 |
| 2012 | 31.0 | 104 | 69.6 | 95.2 | 57 | 43 | 83 |
| 2013 | 105 | 101 | 109 | 106 | 55 | 28 | 79 |
| 2014 | 143 | 111 | 109 | 97.3 | 68 | 35 | 130 |
| 2015 | 84.8 | 107 | 74.4 | 115 | 70.8 | 22.6 | 112.3 |
| 2016 | 43.4 | 107 | 57.2 | 96.5 | 56 | 35 | 90.4 |
| 2017 | 23.6 | 108 | 57.2 | 96.5 | 50 | 26.2 | 69.5 |
| 2018 | 78.0 | 136 | 30.5 | 119 | 56.1 | 34 | 81 |
| 2019 | 69.6 | 121 | 63.8 | 141 | 56.8 | 24.8 | 123.6 |
| ^1^Annual snowfall/precipitation data sourced from John Glenn International Airport (NOAA Station: US1OHFR0052) | | | | | | | |
| ^2^Annual average and high range sodium data sourced from City of Columbus Department of Public Utilities (2007-2020) Dublin Road Water Treatment Plant (City of Columbus Department of Public Utilities, 2007-2020) | | | | | | | |

Table S2. Annual snowfall (cm/yr) and precipitation (cm/yr) data, and annual average/high range sodium concentrations (mg/L) for Columbus, O.H. from 2006-2019^1,2^

| Philadelphia | |  | Pottstown | |  | Havertown | |  |
| --- | --- | --- | --- | --- | --- | --- | --- | --- |
| Sample Date | Na (mg/L) | Cl (mg/L) | Sample Date | Na (mg/L) | Cl (mg/L) | Sample Date | Na (mg/L) | Cl (mg/L) |
| 19-Nov-18 | 50.7 | 95.9 | 18-Nov-18 | NS | NS | 18-Nov-18 | 19.0 | 59.3 |
| 26-Nov-18 | 35.3 | 86.1 | 25-Nov-18 | 21.2 | 23.0 | 25-Nov-18 | 28.7 | 49.7 |
| 2-Dec-18 | 32.9 | 82.2 | 2-Dec-18 | 21.2 | 20.3 | 2-Dec-18 | 22.4 | 39.1 |
| 9-Dec-18 | 35.6 | 93.2 | 9-Dec-18 | 25.1 | 25.2 | 9-Dec-18 | 26.4 | 48.7 |
| 16-Dec-18 | 37.6 | 93.1 | 16-Dec-18 | 21.4 | 26.1 | 16-Dec-18 | 28.1 | 52.6 |
| 23-Dec-18 | 35.3 | 88.2 | 23-Dec-18 | 23.2 | 20.7 | 23-Dec-18 | 31.1 | 33.7 |
| 30-Dec-18 | 31.0 | 85.2 | 30-Dec-18 | 22.3 | 19.6 | 30-Dec-18 | 21.2 | 36.7 |
| 6-Jan-19 | 26.5 | 72.7 | 6-Jan-19 | 20.5 | 18.0 | 6-Jan-19 | 19.9 | 35.0 |
| 13-Jan-19 | 32.5 | 82.4 | 13-Jan-19 | 23.1 | 27.4 | 13-Jan-19 | 24.1 | 42.5 |
| 20-Jan-19 | 47.4 | 118 | 20-Jan-19 | 20.8 | 30.7 | 20-Jan-19 | 27.4 | 49.1 |
| 27-Jan-19 | 72.6 | 163 | 27-Jan-19 | 20.8 | 30.6 | 27-Jan-19 | 26.5 | 48.5 |
| 3-Feb-19 | 75.9 | 165 | 3-Feb-19 | 20.6 | 29.8 | 3-Feb-19 | 39.3 | 76.0 |
| 10-Feb-19 | 62.4 | 140 | 10-Feb-19 | 23.4 | 36.2 | 10-Feb-19 | 45.6 | 87.9 |
| 17-Feb-19 | 114 | 231 | 17-Feb-19 | 32.2 | 63.8 | 17-Feb-19 | 75.1 | 146 |
| 24-Feb-19 | 90.5 | 189 | 24-Feb-19 | 28.6 | 51.4 | 24-Feb-19 | 76.1 | 143 |
| 3-Mar-19 | NS^1^ | NS | 3-Mar-19 | 25.6 | 47.1 | 3-Mar-19 | 50.4 | 98.0 |
| 10-Mar-19 | 127 | 250 | 10-Mar-19 | 26.7 | 51.2 | 10-Mar-19 | 69.2 | 133 |
| 17-Mar-19 | 67.0 | 143 | 17-Mar-19 | 22.5 | 40.1 | 17-Mar-19 | 45.1 | 98.0 |
| 24-Mar-19 | 51.0 | 120 | 24-Mar-19 | 20.2 | 36.0 | 24-Mar-19 | 29.7 | 54.6 |
| 30-Mar-19 | 45.3 | 113 | 31-Mar-19 | NS | NS | 31-Mar-19 | 37.5 | 71.5 |
| ^1^NS = No sample collected on given date | | |  |  |  |  |  |  |

Data Set S1. Sodium and chloride concentrations (mg/L) in tap water for three residences sampled weekly during winter 2018-2019
